# Full left ventricular coverage is essential for the accurate quantification of the area-at-risk by T1 and T2 mapping

**DOI:** 10.1038/s41598-017-05127-0

**Published:** 2017-07-07

**Authors:** Heerajnarain Bulluck, Jennifer A. Bryant, Mei Xing Lim, Xiao Wei Tan, Manish Ramlall, Rohin Francis, Tushar Kotecha, Hector A. Cabrera-Fuentes, Daniel S. Knight, Marianna Fontana, James C. Moon, Derek J. Hausenloy

**Affiliations:** 1 The Hatter Cardiovascular Institute, Institute of Cardiovascular Science, University College London, London, UK; 2 The National Institute of Health Research, University College London Hospitals Biomedical Research Centre, London, UK; 3 Barts Heart Centre, St Bartholomew’s Hospital, London, UK; 4 National Amyloidosis Centre, University College London, Royal Free Hospital, London, United Kingdom; 5 National Heart Research Institute Singapore, National Heart Centre Singapore, Singapore, Singapore; 6 Cardiovascular and Metabolic Disorders Program, Duke-National University of Singapore, Singapore, Singapore; 7 Institute of Biochemistry, Medical School, Justus-Liebig-University, Giessen, Germany; 8 Kazan Federal University, Department of Microbiology, Kazan, Russia; 9 Yong Loo Lin School of Medicine, National University Singapore, Singapore, Singapore

## Abstract

T2-weighted cardiovascular magnetic resonance (CMR) using a 3-slice approach has been shown to accurately quantify the edema-based area-at-risk (AAR) in ST-segment elevation myocardial infarction (STEMI). We aimed to compare the performance of a 3-slice approach to full left ventricular (LV) coverage for the AAR by T1 and T2 mapping and MI size. Forty-eight STEMI patients were prospectively recruited and underwent a CMR at 4 ± 2 days. There was no difference between the AAR_full LV_ and AAR_3-slices_ by T1 (P = 0.054) and T2-mapping (P = 0.092), with good correlations but small biases and wide limits of agreements (T1-mapping: N = 30, R^2^ = 0.85, bias = 1.7 ± 9.4% LV; T2-mapping: N = 48, R^2^ = 0.75, bias = 1.7 ± 12.9% LV). There was also no significant difference between MI size_3-slices_ and MI size_full LV_ (P = 0.93) with an excellent correlation between the two (R^2^ 0.92) but a small bias of 0.5% and a wide limit of agreement of ±7.7%. Although MSI was similar between the 2 approaches, MSI_3-slices_ performed poorly when MSI was <0.50. Furthermore, using AAR_3-slices_ and MI size_full LV_ resulted in ‘negative’ MSI in 7/48 patients. Full LV coverage T1 and T2 mapping are more accurate than a 3-slice approach for delineating the AAR, especially in those with MSI < 0.50 and we would advocate full LV coverage in future studies.

## Introduction

Myocardial salvage index (MSI) is considered a more accurate measure to assess the cardioprotective efficacy of novel therapies aiming to reduce myocardial infarct (MI) size in reperfused ST-segment elevation myocardial infarction (STEMI) patients^1–3^ and it has recently been shown to reduce sample size compared to MI size^3^. To assess MSI, knowledge of both the MI size and the area-at-risk (AAR) are required and can be calculated using the formula MSI = 1 − (MI/AAR). Cardiovascular magnetic resonance (CMR) is considered the gold standard imaging modality for quantifying MI size^4^, and it can also provide information on the edema-based AAR^5–7^. T2-weighted short tau inversion recovery (STIR) imaging in the first week following primary percutaneous coronary intervention (PPCI) has been used to delineate the AAR in reperfused STEMI patients, although its robustness has recently been questioned^8^. T2-mapping^9, 10^ has emerged as a more robust technique for the AAR and native T1 mapping has also been shown to perform well against T2 mapping in a canine model of MI^11^, and in clinical patients at 3.0 T^5^.

The accurate quantification of the AAR conventionally requires full left ventricular (LV) coverage. Recently, a 3-slice approach has been proposed for T2-weighted STIR imaging, with the obvious benefit of shorter scan and analysis time^12^. The main aim of this study was to assess whether the 3-slice approach will also perform as well as full LV coverage, using T1 and T2 mapping to delineate the AAR. Secondly, we aimed to assess the impact of a 3-slice approach for the AAR and MI size on MSI.

## Results

Baseline clinical characteristics of the 48 patients are listed in Table 1. The mean age was 59 ± 13 years and 88% (42/48) male gender. The median onset-to-balloon time was 182 (128–328) minutes. CMR was performed at 4 ± 2 days post PPCI. The mean MI size (using full LV coverage) was 27.4 ± 14.6% LV and late MVO occurred in 63% (30/48) of patients. The average number of T1 and T2 maps for full LV analysis was 8 ± 1 per patient.

**Table 1:** Baseline clinical characteristics of STEMI patients.

Details	Patients with full LV T2 maps	Patients with full LV T1 maps
Number of patients	48	30
Male	40 (83%)	23 (77%)
Age	58 ± 13	55 ± 12
Diabetes Mellitus	9 (19%)	7 (23%)
Hypertension	15 (31%)	8 (27%)
Smoking	15 (31%)	11 (37%)
Dyslipidemia	15 (31%)	8 (27%)
Chest pain onset to PPCI time (minutes)	182 (128–328)	213 (131–385)
Infarct artery (%)		
LAD	28 (58%)	19 (63%)
RCA	18 (38%)	9 (30%)
Cx	2 (4%)	2 (7%)
Pre-PPCI TIMI flow (%)		
0	38 (80%)	21 (70%)
1	1 (2%)	1 (3%)
2	4 (8%)	3 (10%)
3	5 (10%)	5 (17%)
Post-PPCI TIMI flow (%)		
0	1 (2%)	1 (3%)
1	0 (0%)	0 (0%)
2	10 (21%)	2 (7%)
3	37 (77%)	27 (90%)
Timing of CMR/days	4 ± 2	3 ± 1
CMR findings		
LVEF/%	49 ± 8	52 ± 8
LV Mass/g	113 ± 35	121 ± 27
MI size/% LV	27.4 ± 14.6	25.6 ± 14.1
AAR by T2/% LV	42.7 ± 11.9	42.2 ± 11.9

LAD: left anterior descending artery; RCA: right coronary artery; Cx: circumflex artery; TIMI: thrombolysis in myocardial infarction.

### T1 mapping versus T2 mapping, n = 30

In the 30 patients with full LV coverage for T1 and T2 maps, The AAR_full LV_ was not significantly different between the 2 mapping techniques (42.6 ± 12.0% LV versus 42.2 ± 11.9% LV, P = 0.44) with an excellent correlation and agreement on Bland-Altman analysis (R^2^ 0.94, bias: 0.4%, limits of agreement ±5.9%).

### 3-slice versus full LV AAR by T1 mapping, n = 30

There was no difference between the AAR_full LV_ and AAR_3-slices_ by T1 mapping [n = 30, 43 (34–51)% LV versus 40 (32–48)% LV, P = 0.054] and there was a good correlation between the two (R^2^ 0.85). However, there was a small bias of 1.7% LV and the limits of agreement were quite wide at ±9.4% LV.

### 3-slice versus full LV AAR by T2 mapping, n = 48

**Figure 1:**
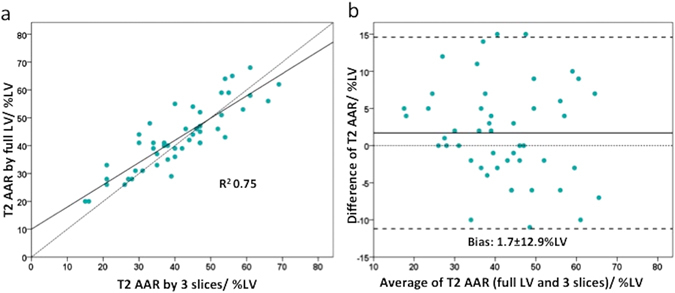
Performance of the full LV coverage versus 3-slice approach for the T2-based AAR. (**a**) The correlation between the 2 approaches; (**b**) The Bland-Altman plot.

Likewise, for T2 mapping, there was no difference between the AAR_full LV_ and AAR_3-slices_ [n = 48, 41 (34–52)% LV versus 40 (32–51)% LV, P = 0.092] and there was a good correlation between the two (R^2^ 0.75) (Fig. 1a). However, there was a small bias of 1.7% LV and the limits of agreement were quite wide at ±12.9% LV (Fig. 1b).

### Full LV coverage MI size versus 3-slice MI size, n = 48

**Figure 2:**
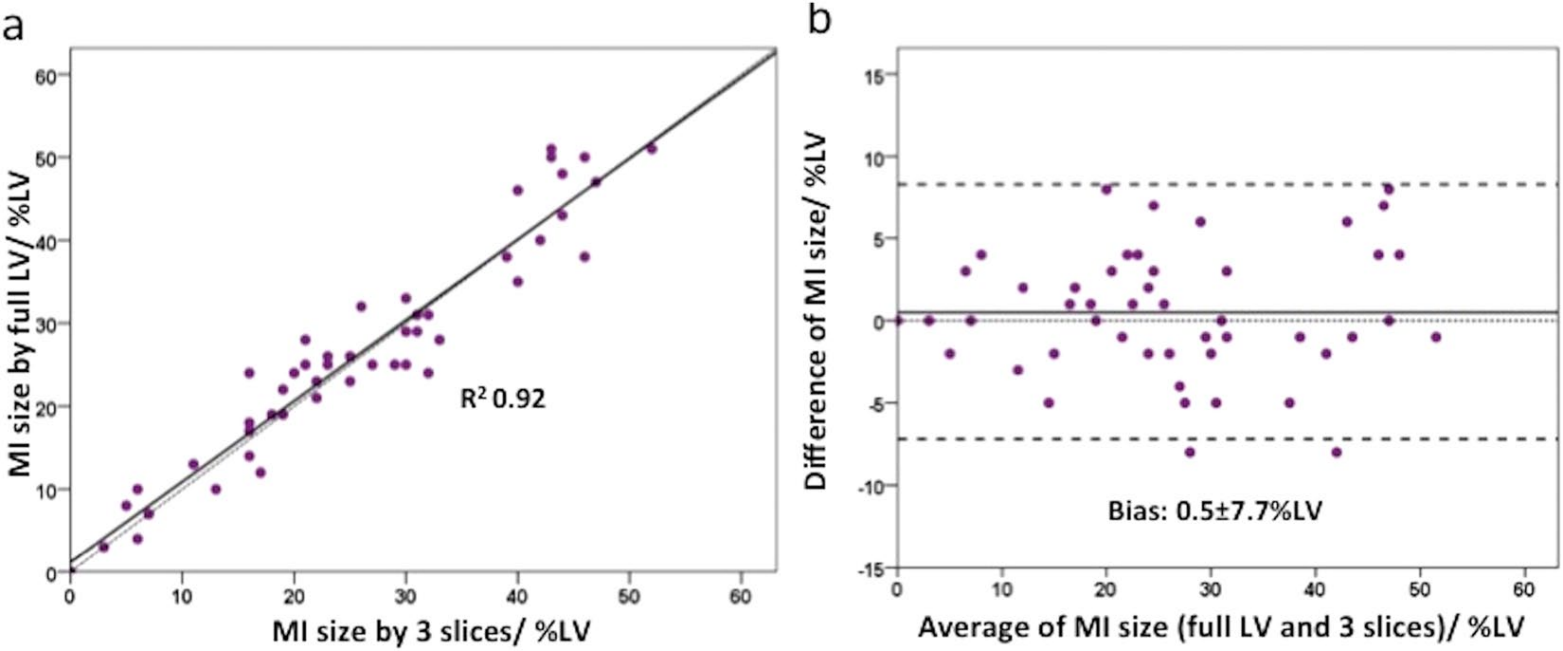
Performance of the full LV coverage versus 3-slice approach for the MI size. (**a**) The correlation between the 2 approaches; (**b**) The Bland-Altman plot.

There was no significant difference between MI size_3-slices_ and MI size_full LV_ (P = 0.93) with a very good correlation between the two (R^2^ 0.92) as shown in Fig. 2a. Bland-Altman analysis showed a small bias of 0.5% but there was a wide limit of agreement of ±7.7% (Fig. 2b).

### MSI: 3-slice (whole LV coverage for MI size and 3 slices for AAR) versus full LV coverage

MSI was calculated using the formula MSI = 1 − (MI size/AAR), where MI size was quantified using full LV short axis coverage and AAR was quantified by using full LV coverage or 3 slices by T2 mapping and T1 mapping.

**Figure 3:**
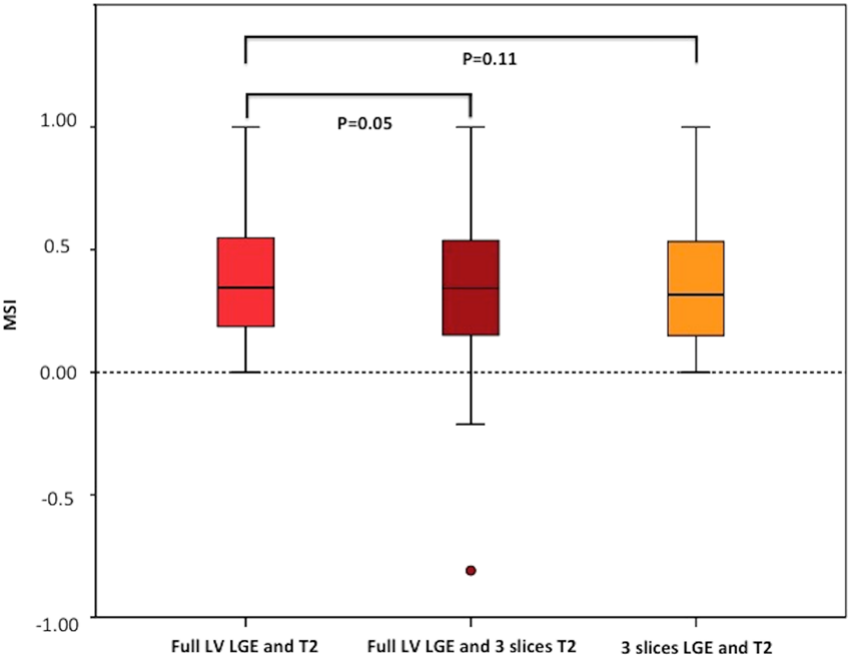
Performance of the 3 approaches for the T2-based MSI. Box and whisker plots of the MSI by T2 using full LV coverage for both the MI size and AAR; 3-slice approach for AAR and full LV coverage for the MI size; and 3-slice approach for both the MI size and AAR. The MSI using 3-slice approach for AAR and full LV coverage for the MI size resulted in negative MSI in 7 patients.

**Figure 4:**
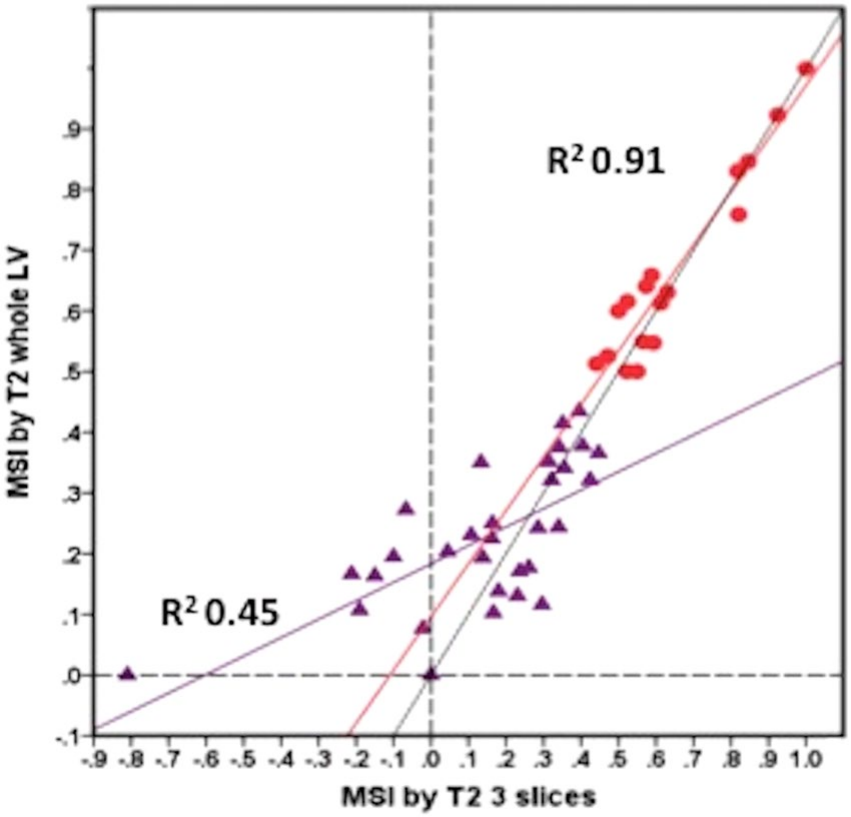
Correlation between the MSI derived by the 2 approaches, subdivided by MSI above or below 0.50. The dotted lines represent the line of identity. The dashed lines represent the lines passing through 0 on the x and y-axis.

For T2 mapping (n = 48), the MSI was 0.35 (0.18–0.55) for T2 AAR_full LV_ and 0.34 (0.14–0.54) for T2 AAR_3-slices_, P = 0.050 (Fig. 3). Although similar, the MSI_3-slices_ performed poorly when the MSI was <0.50 compared to ≥0.50 (R^2^ 0.45 versus R^2^ 0.91, P < 0.001), Fig. 4. Bland-Altman analysis showed a bias of 0.06 but a wide limit of agreement of ±0.33. Furthermore, the 3-slice strategy underestimated the AAR in 7 out of 48 patients and resulted in a negative MSI (Figs 3 and 4). When these 7 patients were excluded, the R^2^ of the remaining 41 patients for MSI_3-slices_ versus MSI_full LV_ was 0.90, with a bias of 0 but limits of agreement of ±0.15, which is still wide for clinical application.

The same pattern was observed for T1 mapping (n = 30). The R^2^ was 0.94 for MSI > 0.50 and was 0.43 for MSI < 0.50 (3 out of 30 patients resulted in a negative MSI using the 3-slice strategy).

### MSI: 3-slice (3 slices for both MI size and AAR) versus full LV coverage, n = 48

**Figure 5:**
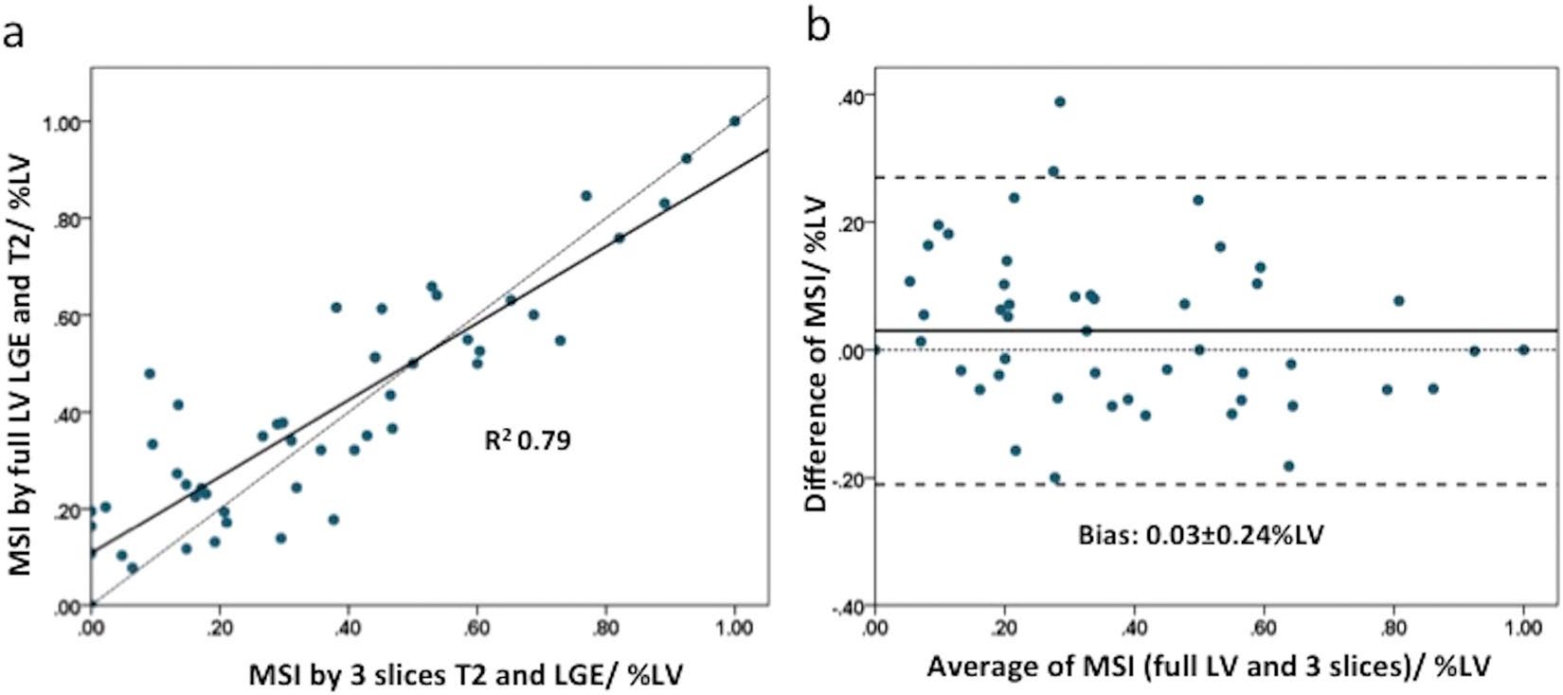
Performance of the full LV coverage versus 3-slice approach (both for the AAR and the MI size) for the MSI. (**a**) The correlation between the 2 approaches; (**b**) The Bland-Altman plot.

When MSI was calculated using 3 slices for both MI size and T2 AAR, there was no significant difference between MSI_full LV_ and the MSI_3-slices_ (P = 0.11) as shown in the box and whisker plots in Fig. 3. There was a moderate correlation between the two (R^2^ 0.79) and Bland-Altman analysis showed a small bias of 0.03 but a wide limit of agreement of ±0.24. Of note, none of the MSI obtained were negative. Visually, there was a wider dispersion between the points and the correlation line for those with MSI < 0.50 than those with MSI > 0.50 in Fig. 5a, which was also reflected in the Bland-Altman plot (Fig. 5b). The R^2^ was 0.28 for those with MSI < 0.50 and 0.65 for those with MSI > 0.50.

## Discussion

These results suggest that although a 3-slice approach had a good correlation with the full LV approach both for the edema-based AAR and MI size, the limits of agreements were quite wide for clinical application and the derived MSI was inadequate, especially when the MSI was <0.50. Furthermore, when MSI was calculated using whole LV coverage for MI size and the 3 slices for AAR as previously done by Hamshere *et al*.^12^, this approach underestimated the AAR in 7/48 patients for T2 mapping and 3/30 patients for T1 mapping and resulted in a negative MSI, which is not plausible in practice and would impact on mean MSI in a cardioprotection study. In the clinical setting, it is difficult to know whether a patient would have MSI more than or less than 0.50 prior to acquiring the images and the analysis the MI size and AAR data and therefore full LV acquisition of T1 or T2 maps is recommended when the edema-based AAR needs to be assessed.

Performing a comprehensive CMR study for research purposes to obtain information on MI size, edema-based AAR, MVO and extracellular volume fraction in a patient with a recent STEMI can take up to 1 hour and therefore shortening the CMR duration is highly desirable. Recently, a 3-slice approach for assessing the AAR by T2-weighted STIR imaging has been shown to perform as well as full LV coverage and offered the possibility to shorten the scan time^12^. However, T2-weighted imaging has several limitations including the subjective interpretation of the images, variations in regional myocardial intensity due to changes in sensitivity of surface coils, blood-pooling artefacts at the subendocardial border, its relatively low contrast-to-noise ratio between normal and abnormal myocardium, and its susceptibility to breathing and motion artefacts^13, 14^. T1 and T2 mapping has recently emerged as a more robust technique to delineate the edema-based AAR^11^ and both these techniques at 1.5 T correlated well with the AAR by single photon emission tomography^15^ and performed equally well against each other in the clinical setting^5^. However, unlike in the study by Hamshere *et al*.^12^, the 3-slice approach did not perform well against full LV approach using T1 and T2 mapping, especially in those patients with MSI < 0.50. Although they included a larger number of patients, the mean MI size and AAR was much smaller in their cohort (MI size: 18% LV versus 27% LV; AAR: 27% LV versus 41% LV) and MSI was larger (41% versus 35%) when compared to our cohort.

### Limitations

Our sample size for comparing 3-slice versus full LV coverage was 48 compared to 85 in the previous study^12^ and no formal power calculation was performed prospectively. However, we used the more robust edema-based AAR technique (both T1 and T2 mapping) and included patients with a range of MI size and AAR. A retrospective power calculation (PASS 15 Power Analysis and Sample Size Software (2017). NCSS, LLC. Kaysville, Utah, USA, ncss.com/software/pass) for paired measurements from a sample size of 48 achieved 100% power to detect non-inferiority using a one-sided t-test when the margin of non-inferiority was set at 0.00% and the true difference between the mean (1.70%) and the standard deviation (6.45%) were derived from the Bland-Altman analysis (significance level (alpha) of 0.15). We did not have T2-weighted STIR images acquired for these patients for comparison.

## Conclusion

Despite the clear benefits of a shorter scan and analysis time, we caution against using a 3-slice approach for the edema-based AAR by T1 and T2 mapping, especially in those with MSI < 0.50. Full LV coverage should remain the quantification approach of choice for the AAR in clinical cardioprotection studies.

## Methods

### Study Population

48 acute STEMI patients reperfused by PPCI from a recently reported cohort were included in this study^16–20^. In brief, the London-Harrow Research Ethics Committee approved this study. These patients were prospectively recruited between August 2013 and July 2014 following informed consent. All research-related procedures were performed in accordance with the local guidelines and regulations. The management of STEMI was as per current guidelines^21^. Study exclusion criteria were known previous MI and standard recognized contraindications to CMR.

### Imaging acquisition

All CMR scans were performed on a 1.5 Tesla scanner (Magnetom Avanto, Siemens Medical Solutions) using a 32-channel phased-array cardiac coil. Full LV coverage native T1 mapping was available in 30 patients. Full LV coverage for T2 mapping and late gadolinium enhancement (LGE) were available in all 48 patients.

#### Native T1 mapping (Work in Progress #448B)

Native T1 maps were acquired with a steady state free precession (SSFP)-based modified Look-Locker inversion recovery (MOLLI) sequence (flip angle = 35°; pixel bandwidth 977 Hz/pixel; voxel size = 1.5 × 1.5 × 6.0 mm; echo time = 1.1 ms; matrix = 256 × 144; slice thickness = 6 mm with 4 mm gap) using a 5s(3s)3s modified sampling protocol^22^. Motion correction and a non-linear least-square curve fitting of the set of images acquired at different inversion times were performed inline by the scanner to generate a pixel-wise colored T1 map^23^.

#### T2 mapping (Work in Progress #448B)

Colored T2 maps consisting of pixel-wise T2 values (Work In Progress 448B, Siemens Healthcare) were generated following motion correction and fitting to estimate T2 relaxation times^9^ after acquiring 3 single-shot images (flip angle = 70°; pixel bandwidth 930 Hz/pixel; voxel size = 2.0 × 2.0 × 6.0 mm; echo time = 1.1 ms; repetition time = 3 × R-R interval; matrix = 116 × 192; slice thickness = 6 mm with 4 mm gap) at different T2 preparation times (0 ms, 24 ms, and 55 ms, respectively).

#### LGE imaging

LGE imaging was acquired with a standard segmented ‘fast low-angle shot’ two-dimensional inversion-recovery gradient echo sequence or a respiratory motion-corrected, free-breathing single shot SSFP averaged phase sensitive inversion recovery sequence^24, 25^ at 10–15 minutes after the injection of 0.1 mmol/kg of Gadoterate meglumine (Gd-DOTA marketed as Dotarem, Guerbet S.A., Paris, France).

### Imaging analysis

All imaging analysis was performed using CVI42 software (Version 5.1.2[303], Calgary, Canada). The imaging analysis methods have been previously described^16–18^. In brief, the endocardial and epicardial borders were manually drawn on the LGE, T1 and T2 maps. Areas of hypo-intense core of microvascular obstruction were included as part of the MI zone and AAR.

For the full LV coverage approach, MI size was quantified using a signal intensity threshold of 5 standard deviations (SD) above the normal remote myocardium^4^ and expressed as a percentage of the whole LV (% LV). All the short axis LGE images covering the whole LV were used to quantify MI size. The full LV edema-based AAR (AAR_full LV_) from the T1 and T2 maps were quantified using a threshold of 2-SD above the remote myocardium and expressed as % LV.

**Figure 6:**
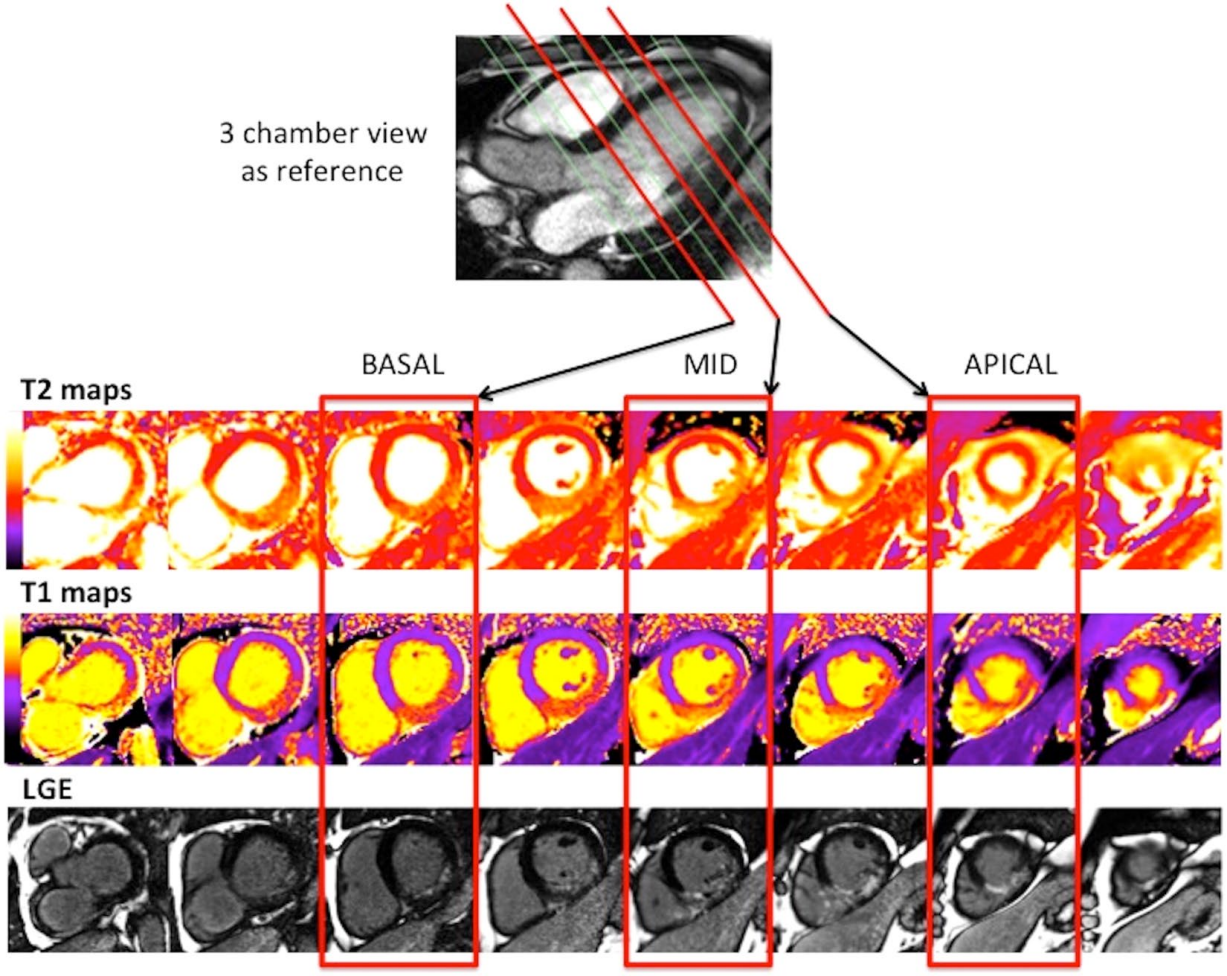
Matching T2 maps, T1 maps and LGE images of a patient with an inferior STEMI. This figure shows the selection of 3 slices (basal, mid and apical) of T2 maps, T1 maps and LGE images from the full LV coverage images of a patient with an inferior STEMI, and with the 3-chamber view as reference.

For the 3-slice approach, only basal, mid and apical LV slices for MI size and AAR were analyzed using the same approach by Hamshere *et al*.^12^ and as illustrated in Fig. 6. In brief, the basal LV slice was chosen as the first short axis slice basally below the LV short axis with left ventricular outflow track. The mid LV slice was the short axis slice with papillary muscle heads visible and at distance of at least 2 slices from the basal LV slice. The apical LV slice was the short axis slice at a distance of at least 2 slices from the mid LV slice and with visible LV cavity present on the short axis. T1, T2 maps and LGE images with matching slice position were used for the AAR_3-slices_ and MI size_3-slices_ quantification.

### Statistical analysis

SPSS version 22 (IBM Corporation, Illinois, US) was used for all statistical analysis. Shapiro-Wilk Test was used to assess for normality. Continuous data was expressed as mean ± standard deviation (SD) or median (interquartile range) and categorical data was reported as frequencies and percentages. Groups were compared using paired Student t-test/Wilcoxon signed rank test or unpaired Student t-test/Mann Whitney U test where appropriate. Correlation was assessed using either Pearson’s correlation coefficient for normally distributed data or Spearman’s rho for non-normally distributed data. Bland-Altman analysis was performed for inter-method agreement and expressed as bias and limits of agreement (±2 SD). All statistical tests were two-tailed, and P < 0.05 was considered statistically significant.
